# GIGANTEA regulates *PAD4* transcription to promote pathogen defense against *Hyaloperonospora arabidopsidis* in *Arabidopsis thaliana*

**DOI:** 10.1080/15592324.2022.2058719

**Published:** 2022-04-04

**Authors:** Anamika Singh

**Affiliations:** a School of Biological Sciences, National Institute of Science Education and Research (Niser) Bhubaneswar, Jatni, India; b Homi Bhabha National Institute, Training School Complex, Mumbai, India; c Department of Plant Developmental Biology, Max Planck Institute for Plant Breeding Research, Cologne, Germany

**Keywords:** *GIGANTEA*, plant defense response, *Hyaloperonospora*, *PAD4*, salicylic acid (SA), basal immunity

## Abstract

Plants have evolved a network of complex signaling pathways that allow them to cope with the fluctuations of internal and external environmental cues. GIGANTEA (GI), a well-known, highly conserved plant nuclear protein, has been shown to regulate multiple biological functions in plants such as circadian rhythm, light signaling, cold tolerance, hormone signaling, and photoperiodic flowering. Recently, the role of GI in disease tolerance against different pathogens has come to light; however, a detailed mechanism to understand the role of GI in pathogen defense remains largely unexplained. Here, we report that *GIGANTEA* is upregulated upon infection with a virulent oomycete pathogen, *Hyaloperonospora arabidopsidis* (*Hpa*), in *Arabidopsis thaliana* accession Col-0. To investigate the role of GI in *Arabidopsis* defense, we examined the pathogen infection phenotype of *gi* mutant plants and found that *gi-100* mutant was highly susceptible to *Hpa* Noco2 infection. Notably, the quantitative real-time PCR showed that *PHYTOALEXIN DEFICIENT4* (*PAD4*) and several PAD4-regulated downstream genes were downregulated upon Noco2 infection in *gi-100* mutant as compared to Col-0 plants. Furthermore, the chromatin immunoprecipitation results show that GI can directly bind to the intronic region of the *PAD4* gene, which might explain the mechanism of GI function in regulating disease resistance in plants. Taken together, our results suggest that *GI* expression is induced upon *Hpa* pathogen infection and GI can regulate the expression of *PAD4* to promote resistance against the oomycete pathogen *Hyaloperonospora arabidopsidis* in *Arabidopsis thaliana*.

## 1. Introduction

Plants are under constant attack from various pathogens. To maintain their propagation, alterations in flowering time have been observed during plant-pathogen interactions. For instance, bacterial pathogens like *Pseudomonas syringae* and *Xanthomonas campestris* and an oomycete, *Peronospora parasitica* infection, cause earlier flowering than uninfected plants in susceptible *Arabidopsis* plants.^1^ In contrast, in *Brassica rapa*, herbivory by the invasive *Spodoptera littoralis* enhances glucobrassicanapin, leading to delayed flowering.^2^ To ensure their survival and reproduction upon pathogen infection, plants induce defense pathways mainly by salicylic acid (SA) and jasmonic acid (JA) signaling pathways. Salicylic acid (SA) signaling-associated mutants such as *SA INDUCTION-DEFICIENT2* (*sid2), eds5*, and *nahG* suppress flowering by elevating the expression of floral repressor *FLOWERING LOCUS C* (*FLC*) gene.^3^ On the other hand, SA regulatory genes like *HOPW1-1-INTERACTING3* (*WIN3*) and *NONEXPRESSOR OF PR GENES1* (*NPR1*) synergistically affect flowering time by altering the expression of flowering regulatory genes, *FLC* and *FLOWERING LOCUS T* (*FT*).^4^ SUMO E3 ligase SIZ1, PLANT U-BOX 13 (PUB13), and MYB30, regulators of SA-mediated defense, have also been documented to regulate flowering time under biotic stress.^5–7^ Like the SA pathway, another defense signaling pathway, jasmonate (JA) signaling, also regulates both negative and positive *Fusarium oxysporum* (*F. oxysporum*) resistance in *Arabidopsis thaliana* (*At*). Upon infection, bHLH transcription factors that suppress JA-mediated defense response promote flowering, while the JA receptor mutant *coi1* shows extreme resistance to *F. oxysporum* and causes early flowering.^8^ Additionally, ethylene (ET)-insensitive mutants cause a delay in flowering time,^9^ whereas HDA6 and HDA19, the histone deacetylases that regulate JA and ET-mediated defense responses, have been shown to enhance the transition to flowering.^10–12^

In plants, the successful transition from vegetative to reproductive growth is a multifaceted trait regulated by a complex network of different genetic pathways, including the vernalization, photoperiod, autonomous, and gibberellin (GA) pathways.^13^ Additionally, several experiments have demonstrated the crucial role of flowering-associated genes in the defense signaling pathway. Regulators of the autonomous pathway, including FPA (an RNA binding protein) and FLOWERING LOCUS D (FLD), promote susceptibility to the bacterial pathogen *Pseudomonas syringae*^14–16^ while *LEAFY*, the floral meristem identity gene, represses key regulators of basal immunity.^17^ The phytohormone GA, which promotes the flowering in *Arabidopsis*,^18^ has also been shown to increase resistance to the bacterial pathogen *Pseudomonas syringae* and confers susceptibility to the necrotrophic fungus *Alternaria brassica*.^19^

GIGANTEA (GI) is one such photoperiodic pathway regulator, and previous studies have shown that GI promotes susceptibility to *F. oxysporum*.^20^ In *Arabidopsis*, mutations in *GIGANTEA* (*gi-1* and *gi-2*) lead to increased resistance to the *F. oxysporum* infection compared to wild-type plants, but the detailed mechanism is unknown.^20^ Throughout numerous stages of plant development, GI plays a role in diverse physiological processes such as flowering time regulation, circadian rhythm, light signaling, starch accumulation, miRNA processing, chlorophyll accumulation, and transpiration.^21–24^ GI has also been shown to regulate abiotic stresses like cold, salt, drought, and oxidative stresses in plants, but its role in pathogen infection remains to be elucidated.^24–29^

Pathogen attack in plants is recognized by innate immune receptors located at the host cell surface or in the cytoplasm. These receptors bind to the conserved microbial molecules (pathogen‐associated molecular patterns, PAMPs) and induce PAMP‐triggered immunity (PTI), which provides early protection from pathogens.^30^ In the course of host-pathogen co-evolution, PTI becomes suppressed by pathogen-derived virulence factors known as effectors to promote infection in the host cell.^31^ These pathogen effectors are primarily sensed by intracellular nucleotide‐binding/leucine‐rich‐repeat (NLR) receptors, which trigger effector‐triggered immunity (ETI). PTI and ETI signaling are associated and increase defense pathways, including mobilization of Ca^2+^‐dependent protein kinase, production of reactive oxygen species (ROS), activation of mitogen‐activated protein kinase (MAPK) signaling cascades, transcriptional reprogramming, and generation of salicylic acid (SA).^32,33^ SA contributes to PTI and ETI, and its biosynthesis upon pathogen recognition is mainly regulated by the SA biosynthetic enzyme gene, *ICS1*.^34–37^ In basal immunity, ENHANCED DISEASE SUSCEPTIBILITY 1 (EDS1) directly binds to PHYTOALEXIN DEFICIENT4 (PAD4) and upregulates *ISOCHORISMATE SYNTHASE 1* (*ICS1*) expression leading to SA accumulation. The downstream events of SA-mediated signaling are executed by the nucleocytoplasmic regulator NONEXPRESSOR OF PR GENES1 (NPR1), a transcriptional co‐activator of SA‐dependent immunity pathways.^38,39^

*Hyaloperonospora arabidopsidis* (*Hpa*) is an obligate biotrophic pathogen of the model plant *Arabidopsis thaliana* and has been extensively used to study host/pathogen co-evolution. Upon infection, it causes downy mildew disease in *Arabidopsis*.^40^ EDS1-PAD4 module activates two branches of immune responses, namely, (i) SA-dependent signaling in which pathogen-induced SA accumulation via *ISOCHORISMATE SYNTHASE 1* (*ICS1*) provides resistance and (ii) SA-independent signaling, which provides resistance via *FLAVIN-DEPENDENT MONOOXYGENASE 1* (*FMO1*).^41–43^ Constitutively overexpressing *FMO1* in *Arabidopsis* revealed enhanced resistance to *Hpa* pathogens, whereas loss-of-function *fmo1* mutants were compromised in resistance to *Hpa*.^41,43,44^ FMO is a pipecolate N-hydroxylase and catalyzes the biochemical conversion of pipecolic acid to N-hydroxypipecolic acid (NHP). NHP systemically accumulates in the plant foliage and induces systemic acquired resistance to pathogen infection.^45,46^

Here, we have explored the role of GIGANTEA (GI) in resistance to biotic stress. Our results indicate that the infection with virulent Noco2 strain of *Hpa* in Col-0 results in increased expression of *GI*. Further, Noco2 infections also lead to increased expression of *PAD4* and its downstream defense signaling genes like *ICS1*, PR1, *FMO1*, and *PBS3*. To confirm our hypothesis that GI mediates the activation of defense signaling pathways, we tested the resistance response of *gi-100* mutant lines against *Hpa* Noco2 infection. In the absence of GI, the pathogen-induced SA-dependent and SA-independent signaling pathways were suppressed leading to increased disease susceptibility. Further, chromatin immunoprecipitation of GI followed by quantitative real-time PCR of *PAD4* shows that GI binds to the intronic region of the *PAD4* gene. On the other hand, expression analysis of *PAD4* in *gi-100* mutant indicates that GIGANTEA positively regulates *PAD4* expression after Noco2 infection. Therefore, our study provides a novel insight into the role of GIGANTEA that likely involves *PAD4*-mediated plant defense responses upon pathogen perception in *Arabidopsis*.

## 2. Material and methods

### 2.1. Plant lines and growth conditions

*Arabidopsis thaliana* lines used in this study were Columbia (Col-0), *eds1-2*, Ws-2, *gi-2* and *gi-100*. Plants were grown on potting soil in a growth chamber at 22°C with 8 h of light (100 μE/m^2^/s) and a relative humidity of 75%.

### 2.2. Growth and infection of downy mildew

*Arabidopsis* plants were grown at 22°C with ∼75% relative humidity (RH) and an 8 h light period. Virulent Noco2 strain of *Hpa* was used for infections. For infection, conidiospore suspensions (5 × 10^4^ conidiospores ml^−1^) were sprayed on 2-week-old *Arabidopsis* seedlings grown on potting soil. Plants were allowed to dry for 1 h and kept at 100% RH for 24 h in a growth chamber with 8 h light at 22°C. Plants were then moved to ∼75% RH for infection to progress, where *Hpa* growth on *Arabidopsis* leaves was scored 6 d post-inoculation by counting spore numbers using a hemocytometer.^47,48^

### 2.3. Microscopy

The infection of Noco2 pathogen in the leaves was visualized by trypan blue staining. Infected leaves were collected in a 1.5-ml centrifuge tube. A 1:1:1:1 volume of lactic acid/glycerol/phenol/H_2_O with trypan blue (1 mg/ml) was added. The tubes were placed in a boiling water bath for 1 min. This was followed by the destaining of leaves in chloral hydrate. The tubes were placed in a speed-vacuum infiltrator for 1 min to remove air bubbles from the leaves.^49^ *Hpa* growth was detected by differential interference contrast microscopy.

### 2.4. RNA extraction and real-time PCR

For RNA isolation, ~50 mg of leaf tissue was harvested in liquid nitrogen and immediately frozen at −80°C. Leaf samples were processed using a Qiagen Plant RNeasy Plant Mini Kit (Cat #74104) according to the manufacturer’s protocol. RNA was quantified using a NanoDrop 1000 spectrophotometer (Thermo Fisher Scientific, USA), and up to 1 μg of RNA was treated with DNase I (TURBO DNA-free kit, Invitrogen) to remove genomic DNA. The cDNA was prepared from 1 µg of RNA of each sample using iScriptTM Reverse Transcription Super-mix (Cat #1708840, Bio-rad, USA). The quantitative RT-PCR (qRT-PCR) was performed using the CFX384TouchTM Real-time detection system (Bio-Rad, USA). Primers used in qRT-PCR were designed using the Primer Quest tool. All reactions were carried out in Hard-shell 384-well PCR plates (supplied by Bio-Rad, Cat #HSP3805), with a reaction volume of 10 µl per well. The PCR mix and thermocycler program for the qRT-PCR were similar to those in Ó’Maoiléidigh et al. 2021.^50^ Transcript levels were normalized with the housekeeping gene, *ACTIN1*. Each qRT-PCR reaction was performed on three biological replicates, and all data were presented as mean ± SEM. Primers used in the qRT-PCR are listed in Supplementary Table 1.

### 2.5. Chromatin immunoprecipitation (ChIP) followed by qPCR

ChIP experiment was performed as published previously^51^ with minor modifications. Three independent biological replicates for Col-0 and 35S::HA:GI were generated. For each sample, 1 g of 10-d-old seedlings were harvested and cross-linked twice by infiltration with 1% formaldehyde under a vacuum for 10 minutes. The material was collected from plants grown in LDs at 22°C for 10 days (16 hlight and 8 h dark). Nuclei were disrupted by sonication four times for 5 min in cycles of 15 sec “on”/15 sec “off,” with a 1 min incubation between each sonication treatment using a water bath Bioruptor (Diagenode). Chromatin immunoprecipitation was performed by using HA antibodies (Abcam, ab9110) and Protein A agarose beads (11719408001, Roche). This was followed by DNA precipitation using 3 mM sodium acetate and absolute ethanol. DNA pellets were washed with 70% ethanol, air-dried, and resuspended in water before qRT-PCR. A small aliquot of untreated sonicated chromatin was used as the total input DNA. ChIP-qPCR was analyzed, and the relative enrichment of the IP/Input was normalized to *ACTIN8*. Primers were designed from the exon 2 region, intron 1 region and transcription start site (TSS) of the *PAD4* gene, and qRT-PCR was performed in three biological replicates. Primers used in the ChIP-qPCR are listed in Supplementary Table 2.

## 3. Results

### 3.1. *Loss-of-function mutant*, gi-100 *shows an enhanced susceptibility to* Hpa *Noco2 infection*

**Figure 1.**
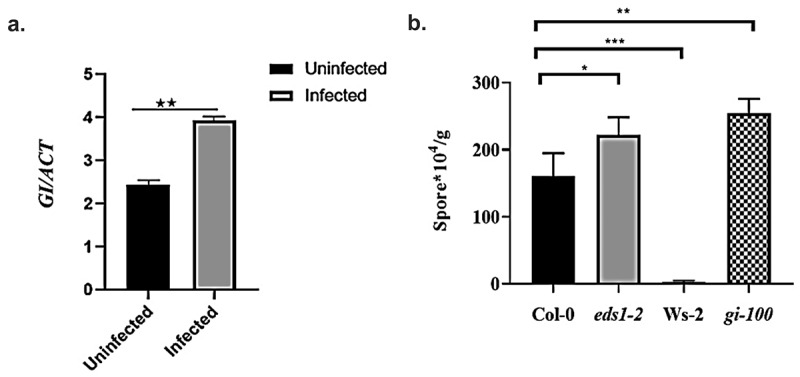
Involvement of GIGANTEA in regulating Noco2 infection in *Arabidopsis*. (a) Relative abundance of *GI* transcripts in uninfected (0 hpi) and infected (48 hpi) Col-0 plants using qRT-PCR. Transcripts levels were normalized with the internal control gene, *ACTIN1*. Three biological replicates were used for the experiments. Data represented as Mean ± SE. Student’s paired T-test was used to evaluate the significant differences as *p < .05. ** p < .01, *** p < .001 compared between uninfected and infected Col-0 plants. (b) Graph showing conidiospores count (*10^4^/g) after 6 d of Noco2 infection in Columbia (Col-0), *eds1-2*, Ws-2 and *gi-100*. Plants were grown on potting soil in a growth chamber at 22°C with 8 h of light and relative humidity of 75%. For Noco2 infections, 2-weeks-old *Arabidopsis* seedlings of the indicated genotype were infected with conidiospores suspension (5 × 10^4^ conidiospores/ml) and oomycete sporulation was measured using hemocytometer at 6 dpi. Three biological replicates were used for the experiments. To test for significance among the dataset, a one-way analysis of variance (ANOVA) followed by Sidak’s multiple comparisons test was performed using GraphPad prism software at *p < .05. ** p < .01, *** p < .001.

To explore the function of GI in activating defense during plant-biotrophic pathogen interaction, we first evaluated the enrichment of *GI* expression upon pathogen infection using downy mildew pathogen *Hyaloperonospora arabidopsidis* (*Hpa*). The most common symptom for this obligate biotrophic pathogen is the aerial conidiophores. Here, we have used virulent Noco2 strain of *Hpa* for infection. To investigate the possible alteration in *GI* expression, transcript abundance was analyzed by qRT-PCR in the Col-0 leaf sample after 48 h of infection with Noco2 strain. An approximately >1.5-fold increase in the level of *GI* transcripts was observed compared to its uninfected control (Figure 1a). This result revealed that *GI* is a pathogen-inducible gene (*Hpa* Noco2).

To further confirm the role of GI as a resistance factor during plant-biotrophic pathogen interactions, the well-characterized GI T-DNA insertional null mutant *gi-100*^52^ and the wild-type Col-0 plants were challenged with *Hpa* Noco2. It is known that Ws-2 plants show resistance to Noco2 strain due to the presence of functional RPP1 receptor proteins and hence were used as a negative control for this experiment.^53^ EDS1 acts as a positive regulator of plant defense signaling, while its absence makes the plant more susceptible to pathogens, and hence, *eds1-2* mutant plants were used as a positive control.^42^ The growth of *Hpa* is estimated in two ways: counting conidiospores^54^ or counting sporangiophores after trypan blue staining.^55^ Plants were infected with the *Hpa*, Noco2 strain (10^4^ conidiospores per milliliter), which is virulent on Col-0, and the appearance of conidiospores was scored 6 d later using a hemocytometer. We observed that *gi-100* mutant plants were highly susceptible to Noco2 infection than the wild-type Col-0 control (Figure 1b). The infection was further evaluated by trypan blue staining of the conidiospores, which confirmed the higher colonization of conidiospores in *gi-100* mutant compared to Col-0 (Supplementary Figure S1). Hence, the *gi-100* mutant demonstrated enhanced susceptibility to the *Hpa* Noco2 infection in comparison to Col-0 plants. Thus, our results confirmed the positive role of GI in conferring pathogen resistance during plant-pathogen interaction. We have also checked the Noco2 fungal infection severity using another well-characterized *gi* mutant, *gi-2*.^56^ Similar to *gi-100, gi-2* also cause increased susceptibility to *Hpa* infection; however, *gi-2* mutant has a weaker infection phenotype than *gi-100*. The trend of spore count after Noco2 infection in *gi-2* was in the same direction as *gi-100* when compared to Col-0. But, unlike *gi-100* mutant plants, *gi-2* mutant could not surpass the *eds1-2* mutant spore count number after infection (Supplementary Figure S3). This difference in the severity of phenotypes could be because both the *gi* mutants, *gi-2* and *gi-100*, have been shown to form different sizes of transcripts after T-DNA insertion.^24^

### 3.2. *GI binds to the intronic region of* PAD4

**Figure 2.**
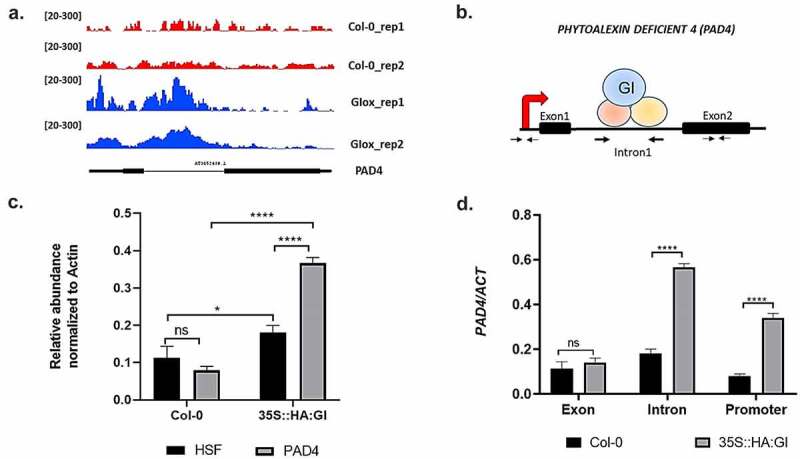
Chromatin immunoprecipitation assay to show binding of GI to *PAD4*. (a) Visualization of GI ChIP-seq data in the genomic region encompassing the *PAD4* locus. Peaks represent the sequence enrichment of *PAD4* in Col-0 (red) and GI-overexpressed (blue) lines. (b) Schematic picture showing the regions of the *PAD4* gene from which the primers were designed. (c) Graph showing enrichment of *PAD4* gene after qRT-PCR using chromatin isolated from plants expressing HA-tagged GI (35S::HA:GI) and wild type (Col-0). Immunoprecipitation was done using HA-beads followed by qPCR. Gene expression was normalized to the internal housekeeping *ACTIN8* gene. In this experiment, Heat shock protein (*HSF*) gene was used as a negative experimental control to show that binding of GI to *PAD4* is specific and it is not binding to any random gene sequences. (d) qRT-PCR analysis of different regions of *PAD4* gene using HA pull-down chromatin samples from Col-0 and 35S::HA:GI. Primers were designed from the exon 2 region, intron 1 region, and transcription start site (TSS) of the *PAD4* gene. Three biological replicates were used for the experiment. To test for significance among the dataset, a two-way analysis of variance (ANOVA) followed by Tukey’s multiple comparisons test was performed using GraphPad prism software at *p < .05. ** p < .01, *** p < .001.

We further explored the detailed mechanism by which GI confers resistance against *Hpa* pathogen infection. In a study by Nohales et al. 2019, putative GI binding target sites were identified by chromatin immunoprecipitation sequencing (ChIP-seq) using a pull-down of GI protein from the *Arabidopsis* chromatin preparation. To know the potential immunity pathway targets of GI, we explored the ChIP-seq data available online^57^ and identified *PHYTOALEXIN DEFICIENT4* (*PAD4*) as one of the putative targets of GI (Figure 2a). Several studies show that *PAD4* along with the *EDS1* regulates plant basal immunity against virulent biotrophic pathogens.^42^ It is documented that the regulatory sequences of the *PAD4* gene are present in the promoter as well as the intronic region of the gene and these regulatory sequences of *PAD4* contain a G‐box element.^58^ G-box-like elements are considered as potential binding sites of GI.^57^ Based on the above pieces of evidence, we hypothesized that GI functions upstream of *PAD4* and might regulate *PAD4* by binding to the G-box element present in the intronic regulatory sequences.

In order to test our hypothesis, we performed chromatin immunoprecipitation followed by quantitative RT-PCR (ChIP-qPCR). Chromatin was isolated from plants expressing HA-tagged GI protein (35S::HA:GI) and control Col-0 plants, followed by chromatin fragmentation, and then immunoprecipitation was performed using an anti-HA antibody. Here, Col-0 plants were used as a negative control to ensure the specific binding of proteins with anti-HA antibody only. Pull-down was further followed by qPCR from the purified DNA fragments using primers specific to the intronic sequences of the *PAD4* gene. Exon-specific primers and Transcription start site (TSS)-specific primers of the *PAD4* gene were used as a control to show the specific binding of GI to *PAD4* intron in comparison to promoter and exon regions. Our ChIP‐qPCR result detected the highest enrichment of *PAD4* in the intronic region followed by the promoter region of the *PAD4* while binding to the exon region was not significant. In this experiment, heat shock protein (*HSF*) gene was used as a negative experimental control to show that binding of GI to *PAD4* is specific and it is not binding to any random gene sequences. The samples were normalized with *ACTIN8* as an internal control. Thus, our results confirmed that GI specifically binds to the intronic region of *PAD4* (Figure 2c and d).

**Figure 3.**
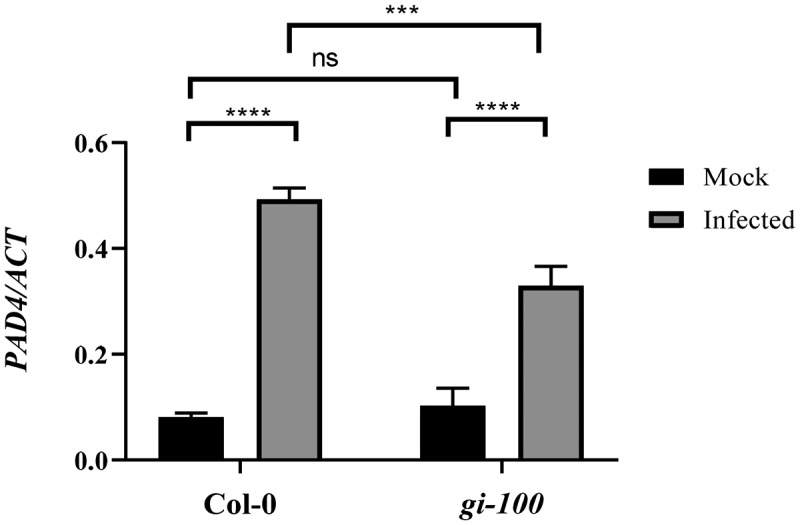
Regulation of *PAD4* expression by GI after Noco2 infection. Graph showing qRT-PCR result of *PAD4* gene expression relative to *ACTIN1* gene at 48 hpi. Three biological replicates were used for the experiments. Data represented as Mean ± SE. To test for significance among the dataset, a two-way analysis of variance (ANOVA) followed by Tukey’s multiple comparisons test was performed using GraphPad prism software at *p < .05. ** p < .01, *** p < .001.

After confirming that GI can physically associate at the regulatory sequences of the *PAD4* gene, we wanted to explore whether changes in *GI* expression can regulate *PAD4* gene expression during *Hpa* infection. The amount of *PAD4* expression was measured using quantitative RT-PCR in Col-0 and *gi-100* mutant plants, 48 h after inoculation with the virulent Noco2 pathogen. As expected, pathogen‐infected WT plants displayed much higher levels of *PAD4* mRNA expression in comparison to uninfected plants. However, in the case of *gi-100* mutant plants, the *PAD4* expression level was much reduced in comparison to wild-type Col-0 plants upon infection (Figure 3). The above observation suggested that GI is required for the expression of *PAD4* gene in *Arabidopsis* during Noco2 pathogen-induced infection.

### 3.3. GIGANTEA promotes SA-dependent defense genes after infection

**Figure 4.**
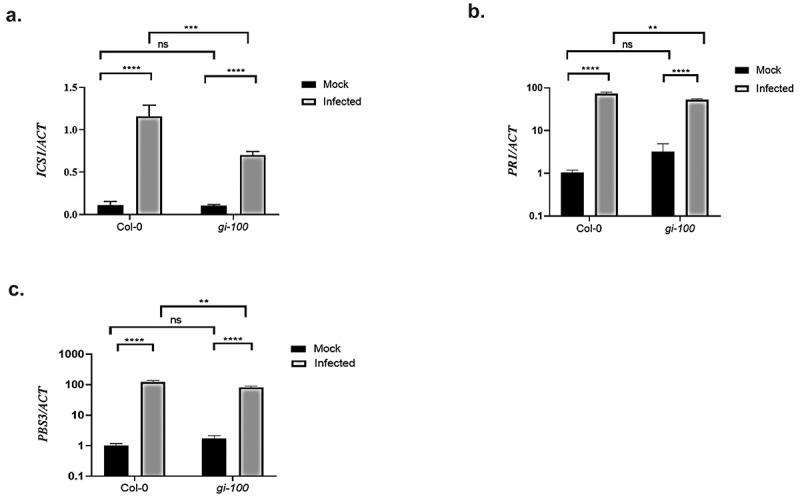
Transcripts accumulation of key genes involved in the Salicylic acid pathway during *Hpa* Noco2 infection. Relative abundance of *ICS1* (a), *PR1* (b) and *PBS3* (c) transcripts, derived from qRT-PCR, after 48 h of Noco2 inoculation in *gi-100* as well as Col-0 lines of *Arabidopsis*. Values are expressed as mean ± SD. The Y-axis represents the 2^−^^^CT^ of *ICS1* gene and log10 (2^−^^^^CT^) of *PR1 and PBS3* transcription. Transcripts levels were normalized with the internal control gene, *ACTIN1*. Three biological replicates were used for the experiments. To test for significance among the dataset, a two-way analysis of variance (ANOVA) followed by Tukey’s multiple comparisons test was performed using GraphPad prism software at *p < .05. ** p < .01, *** p < .001.

After confirming the role of GI in activating *PAD4* expression to confer pathogen resistance, we further investigated the status of PAD4 downstream signaling pathways that are involved in the disease resistance. Salicylic acid (SA) is the universal component responsible for conferring resistance against biotrophic pathogens. Therefore, we checked the transcription status of SA marker gene, *PR1* and genes involved in SA production, *ICS1* and *AVRPPHB SUSCEPTIBLE3* (*PBS3*). Quantitative RT-PCR was performed using Col-0 and *gi-100* plant samples after 48 h post-Noco2 infection (hpi). A significant decrease in the transcript level of *ICS1*, PR1, and *PBS3* was recorded in the *gi-100* mutant in comparison to the Col-0 plants upon Noco2 infection (Figure 4a–c), indicating that in the absence of *GI*, pathogen-induced expression of SA pathway components is compromised after *Hpa* Noco2 infection.

### 3.4. GIGANTEA promotes SA-independent defense genes after infection

**Figure 5.**
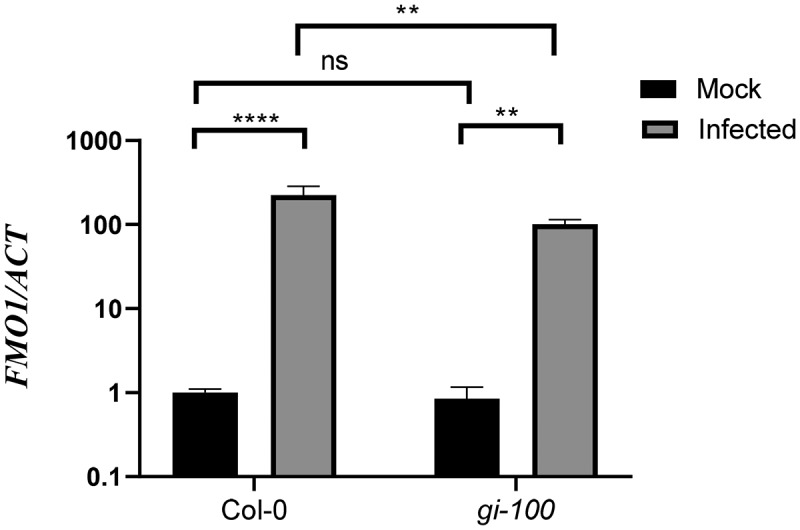
Function of GIGANTEA in activation of SA-independent defense genes after Noco2 infection. Graph showing transcripts accumulation of *FMO1* gene which is involved in SA-independent defense pathway after 48 h of Noco2 infection in *gi-100* as well as Col-0. Values are expressed as mean ± SD. The Y-axis represents the log10 (2^−^^^^CT^) of *FMO1* transcription. Transcripts levels were normalized with the internal control gene, *ACTIN1*. Three biological replicates were used for the experiments. To test for significance among the dataset, a two-way analysis of variance (ANOVA) followed by Tukey’s multiple comparisons test was performed using GraphPad prism software at *p < .05. ** p < .01, *** p < .001.

In addition to the SA-dependent pathway, EDS1 and PAD4 cooperate to activate the expression of SA-independent signaling genes upon pathogen infection. *FLAVIN-DEPENDENT MONOOXYGENASE 1* (*FMO1*), marker genes for SA-independent pathway, is expressed downstream of *EDS1*- and *PAD4*-mediated defense signaling and is required for basal resistance to invasive virulent pathogens.^41,44^ Here, we quantified *FMO1* expressions in Col-0 and *gi-100* mutant lines after infection with Noco2, by qRT-PCR in relation to a constitutive reference gene, *ACTIN1*. This analysis revealed that the expression level of *FMO1* transcripts was significantly reduced in the *gi-100* mutant in comparison to Col-0 plants upon Noco2 infection (Figure 5a). Thus, as a result of compromised activation of the disease-resistance genes in the absence of GI, the *gi-100* mutant plants are more susceptible to the Noco2 infection. Taken together, our findings provide a strong indication for a previously unknown function of GI in conferring disease resistance during *Hpa* Noco2 infection.

**Figure 6.**
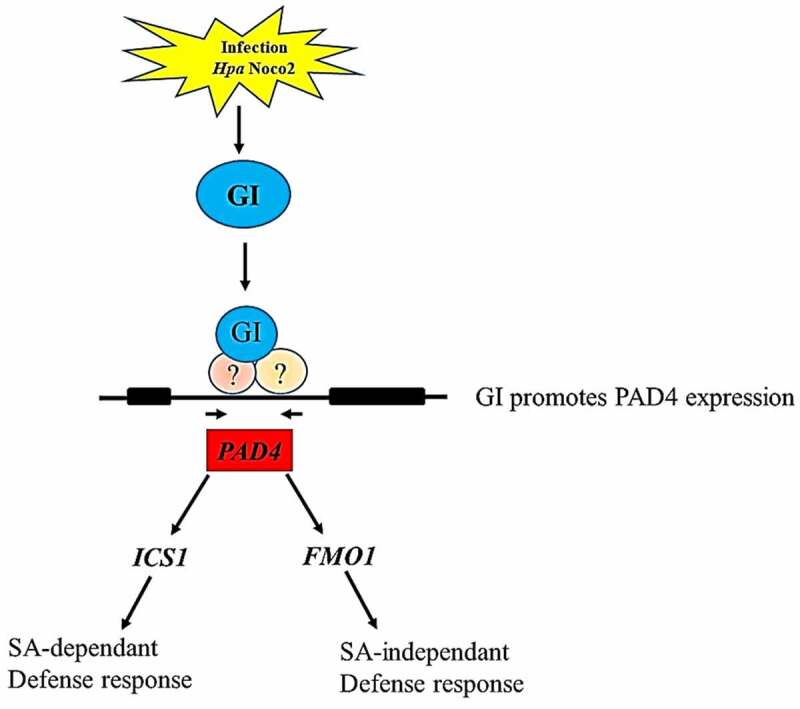
A concluding model depicting the role of GI in regulating *PAD4* expression which is a key player involved in modulating both SA-dependent and SA-independent pathways. Our result shows that *GI* expression is induced in response to *Hpa* Noco2 infection that contributes to enhanced expression of the *PAD4* gene. Graphical summary of the GI-mediated immune response against Hyaloperonospora arabidopsidis infection (Figure 6). Activated *PAD4* further induces its downstream signaling genes expression to initiate the immune response against biotrophic pathogens infection by activating SA-dependent and SA-independent signaling pathways.

## Discussion

GIGANTEA (GI) is a plant-specific nuclear protein and has been shown to be involved in the regulation of many physiological and developmental processes in plants.^24^ Recent studies have revealed a link between GIGANTEA and plant defense signaling.^20,59^ It was observed that late-flowering mutants are immune to the *Fusarium* infection and the absence of GI makes the plant more resistant to the *Fusarium* pathogen.^20^ Another recent publication showed the role of GI as a negative regulator of plant defense signaling during hemibiotrophic fungal infection and confirmed that GI causes susceptibility to *Bipolaris sorokiniana* in *Arabidopsis*.^59^ Based on our results, we proposed a new model where GI may act as a positive regulator of defense signaling and can be a resistance factor for downy mildew disease, which is caused by an obligate biotrophic pathogen, *Hyaloperonospora arabidopsidis* (*Hpa*). Our studies show an additional layer of transcriptional regulation of the plant-pathogen defense signaling pathway. In *gi-100* mutant, upon *Hpa* Noco2 infection, the formation of aerial conidiospores is significantly higher than the Col-0 (control), *eds1-2* mutant (susceptible plant to Noco2 infection) and Ws-2 (an accession resistant to Noco2 infection). This suggests that GI plays a positive role in defense signaling against *Hpa* Noco2 infection and the absence of GI weakens this defense against biotrophic fungus pathogens. We have also investigated the *Hpa* Noco2 fungal infection severity using another *GI* T-DNA insertional mutant line, *gi-2*.^56^ Our results established that *gi-2* shows a similar but comparatively weaker phenotype than *gi-100* upon infection. The *gi-2* mutant lines were more susceptible to *Hpa* Noco2 infection in comparison to Col-0 but could not surpass *eds1-2* mutant phenotype. This difference in the severity of phenotypes could be because both the *gi* mutants, *gi-2* and *gi-100*, have been shown to form different sizes of transcripts after T-DNA insertion,^24,56^ and there could be a difference in the functionality of truncated proteins formed in both the mutants. There are previous reports that have shown a difference in phenotype severity in different alleles of the same gene. For instance, the *gi-1* mutation shortened period lengths of leaf movement, *cab2::1uc* luminescence and RNA transcript abundance rhythms; in contrast, *gi-2* caused a gradual lengthening of the *cab2::1uc* luminescence and RNA transcript abundance rhythm along with shortened period lengths leaf movement.^21^ In another example, the period of *gi-3* did not display wild-type phenotype at 17°C unlike the *gi-11* mutant.^60^ Also, the *gi-2* allele shows an exception in the temperature-independent flowering phenotype of *gi* mutants.^61^ Thus, such variations in different *gi* mutant phenotypes resulting from differences in the functionality of truncated transcripts could also explain the difference in the observed pathogen-related phenotype in our current study. Another possible explanation for variations in disease severity phenotype of *gi-100* and *gi-2* could be that the expression of *GI* neighboring genes is slightly altered in *gi-100* mutant seedlings^52^ and our data could not rule out the contributions from the altered expression of the neighboring genes in plant defense signaling.

Our results also suggest the context-dependent function of GIGANTEA in plant disease resistance. During hemibiotrophic vascular infections like *Fusarium oxysporum* or *Bipolaris sorokiniana*, GI functions as a negative regulator and makes the plant more susceptible to the disease.^20,59^ On the other hand, during downy mildew infection caused by the obligate biotrophic pathogen *Hpa*, GI acts as a positive regulator. To keep the host cell alive, biotrophic pathogens and hemibiotrophs in their biotrophic stage delay senescence. The host can achieve resistance by activating senescence-like processes. On the other hand, necrotrophic pathogens and hemibiotrophs in their necrotrophic stage induce senescence in the host and preventing early senescence is a resistance approach of plants.^62^ This difference in the mode of action could explain the contrasting phenotypes of *gi* mutants with these two pathogens, *Fusarium* and *Hpa*.

Like humans, plants have developed a very effective system of immunity, which enables them to protect themselves from infection and to produce seeds successfully. Several studies demonstrate that PAD4 along with the EDS1 forms one of the core components to regulate plant basal immunity against virulent biotrophic pathogens.^42^ We hypothesized that the defense responses regulated by GI might also involve a PAD4/EDS1 signaling cascade. It has been shown that the regulatory region of the *PAD4* gene lies within the intron, which contains the core ACGT sequences, called G-box sequences, that are required for binding of the bZIP class of transcription regulators, such as GBF1.^58^ GBF1 is a well-established regulator of plant defense signaling and modulates *PAD4* expression.^58^ It has been speculated that GI can also be a G-box binding protein;^57^ however, there is no concrete evidence to date. Based on the above-mentioned findings, we hypothesized that GI can bind to the regulatory intronic sequence of the *PAD4* gene to direct its expression just like GBF1. To confirm our hypothesis, we explored the ChIP-seq results of GI available online,^57^ and to our surprise, we found out that GI can directly be involved in the transcriptional regulation of *PAD4*. We validated this finding with the ChIP assay followed by qPCR. We are first to confirm that GI binds to the regulatory region present in the intron of the *PAD4* gene. Although there is another non‐recognized G‐box (TACGTA) present in the *PAD4* promoter about 1.26 kb upstream of the transcription start site, however, we found less enrichment of the PAD4 start site than the intronic region after pull-down with GI. However, whether GI and GBF1 function together or independently to regulate *PAD4* expression remains an open question. It might be possible that GI along with GBF1 forms a complex that binds to the intronic region of *PAD4* to regulates the plant defense responses. Here, we have confirmed that GI is required for the upregulation of *PAD4* expression during Noco2 infection as *gi-100* mutant showed a reduction in the expression of *PAD4* in comparison to the WT plants. Since *PAD4* expression was not completely blocked in the *gi-100* mutant, it led us to propose that several other alternative regulators (such as GBF1) may function together or independent of GI to fine-tune the expression of *PAD4*, thereby regulating plant defense response to various pathogens.

After confirming the role of GI in the regulation of *PAD4* expression, we next explored the status of PAD4 downstream signaling events. EDS1 and PAD4 are important regulators of basal resistance to obligate biotrophic and certain hemibiotrophic pathogens, governing the accumulation of the phenolic signaling molecule salicylic acid.^43,63^ The EDS1-PAD4 complex promotes the expression of key genes involved in SA biosynthesis, *ICS1* and *PBS3* genes, and SA marker gene, *PR1*.^38^ Our results demonstrated that following the reduced *PAD4* expression in *gi-100* plants after Noco2 infection, PAD4‐dependent defense responses were also faded. Here, we found that after infection, the absence of GI leads to reduced expression of *ICS1, PBS3* and *PR1*. Hence, in wild-type plants, *Hpa* infection results in increased *GI* expression, which enhances the *PAD4* expression, leading to the upregulation of downstream defense genes *ICS1, PBS3*, and *PR1* genes, and hence makes the wild-type plant relatively more resistant to Noco2 infection than *gi-100* plants.

EDS1 and PAD4 also function through the SA-independent mechanism that offers resistance in the presence of FLAVIN-DEPENDENT MONOOXYGENASE 1 (FMO1).^41–43^ *FMO1* is the marker gene of the EDS1-PAD4-controlled, SA-independent signaling pathway. It has been shown that its expression is being locally and systemically stimulated in *Arabidopsis* plants upon inoculation with virulent or avirulent *Pseudomonas syringae* bacteria and oomycete, *Hyaloperonospora arabidopsidis* pathogens, whereas *fmo*1 loss-of-function mutants lead to compromised resistance to virulent or avirulent *P. syringae* or *H. arabidopsidis*.^41,44^ Our results verified that the absence of GI leads to downregulation of *FMO1* after *Hpa* Noco2 infection, hence making the plant more susceptible than Col-0 plants. Further studies in this direction are required to unravel how GI regulates pattern-triggered and the effector‐triggered immune response in *Arabidopsis*.

In summary, *GI* expression is induced in response to *Hpa* Noco2 infection that contributes to enhanced expression of the *PAD4* gene. Activated *PAD4* further induces its downstream signaling gene expression to initiate the immune response against biotrophic pathogen infection by activating SA-dependent and SA-independent signaling pathways. Thus, our results provide a framework that addresses the role of *GIGANTEA* in regulating plant defense response.

## Supplementary Material

Supplemental Material

## References

[1] Korves TM, Bergelson J. A developmental response to pathogen infection in *Arabidopsis*. Plant Physiol. 2003;133:339–10. doi:10.1104/pp.103.027094. 12970499 PMC196610

[2] Schiestl FP, Kirk H, Bigler L, Cozzolino S, Desurmont GA. Herbivory and floral signaling: phenotypic plasticity and tradeoffs between reproduction and indirect defense. New Phytol. 2014;203:257–266. doi:10.1111/nph.12783. 24684288

[3] Martínez C, Pons E, Prats G, León J. Salicylic acid regulates flowering time and links defence responses and reproductive development. Plant J. 2004;37:209–217. doi:10.1046/j.1365-313X.2003.01954.x. 14690505

[4] Wang GF, Seabolt S, Hamdoun S, Ng G, Park J, Lu H. Multiple roles of WIN3 in regulating disease resistance, cell death, and flowering time in *Arabidopsis*. Plant Physiol. 2011;156:1508–1519. doi:10.1104/pp.111.176776. 21543726 PMC3135961

[5] Lee J, Nam J, Park HC, Na G, Miura K, Jin JB, Yoo CY, Baek D, Kim DH, Jeong JC, et al. Salicylic acid-mediated innate immunity in *Arabidopsis* is regulated by SIZ1 SUMO E3 ligase. Plant J. 2007;49:79–90. doi:10.1111/j.1365-313X.2006.02947.x. 17163880

[6] Li W, Ahn IP, Ning Y, Park CH, Zeng L, Whitehill JG, Lu H, Zhao Q, Ding B, Xie Q, et al. The U-Box/ARM E3 ligase PUB13 regulates cell death, defense, and flowering time in *Arabidopsis*. Plant Physiol. 2012;159:239–250. doi:10.1104/pp.111.192617. 22383540 PMC3366716

[7] Liu L, Zhang J, Adrian J, Gissot L, Coupland G, Yu D, Turck F. Elevated levels of MYB30 in the phloem accelerate flowering in *Arabidopsis* through the regulation of FLOWERING LOCUS T. PLoS One. 2014;9:e89799. doi:10.1371/journal.pone.0089799. 24587042 PMC3934951

[8] Song S, Qi T, Fan M, Zhang X, Gao H, Huang H, Wu D, Guo H, Xie D. The bHLH subgroup IIId factors negatively regulate jasmonate-mediated plant defense and development. PLoS Genet. 2013;9:e1003653. doi:10.1371/journal.pgen.1003653. 23935516 PMC3723532

[9] Ogawara T, Higashi K, Kamada H, Ezura H. Ethylene advances the transition from vegetative growth to flowering in *Arabidopsis thaliana*. J Plant Physiol. 2003;160:1335–1340. doi:10.1078/0176-1617-01129. 14658386

[10] Zhou C, Zhang L, Duan J, Miki B, Wu K. HISTONE DEACETYLASE19 is involved in jasmonic acid and ethylene signaling of pathogen response in *Arabidopsis*. Plant Cell. 2005;17:1196–1204. doi:10.1105/tpc.104.028514. 15749761 PMC1087996

[11] Wu K, Zhang L, Zhou C, Yu CW, Chaikam V. HDA6 is required for jasmonate response, senescence and flowering in *Arabidopsis*. J Exp Bot. 2008;59:225–234. doi:10.1093/jxb/erm300. 18212027

[12] Yu CW, Liu X, Luo M, Chen C, Lin X, Tian G, Lu Q, Cui Y, Wu K. HISTONE DEACETYLASE6 interacts with FLOWERING LOCUS D and regulates flowering in *Arabidopsis*. Plant Physiol. 2011;156:173–184. doi:10.1104/pp.111.174417. 21398257 PMC3091038

[13] Andrés F, Coupland G. The genetic basis of flowering responses to seasonal cues. Nat Rev Genet. 2012;13:627–639. doi:10.1038/nrg3291. 22898651

[14] Lyons R, Iwase A, Gänsewig T, Sherstnev A, Duc C, Barton GJ, Hanada K, Higuchi-Takeuchi M, Matsui M, Sugimoto K, et al. The RNA-binding protein FPA regulates flg22-triggered defense responses and transcription factor activity by alternative polyadenylation. Sci Rep. 2013;3:2866. doi:10.1038/srep02866. 24104185 PMC3793224

[15] Singh V, Roy S, Giri MK, Chaturvedi R, Chowdhury Z, Shah J, Nandi AK. *Arabidopsis thaliana* FLOWERING LOCUS D is required for systemic acquired resistance. Mol Plant Microbe Interact. 2013;26(9):1079–1088. doi:10.1094/MPMI-04-13-0096-R. 23745676

[16] Singh V, Roy S, Singh D, Nandi AK. *Arabidopsis* FLOWERING LOCUS D influences systemic-acquired-resistance- induced expression and histone modifications of WRKY genes. J Biosci. 2014;39:119–126. doi:10.1007/s12038-013-9407-7. 24499796

[17] Winter CM, Austin RS, Blanvillain-Baufumé S, Reback MA, Monniaux M, Wu MF, Sang Y, Yamaguchi A, Yamaguchi N, Parker J, et al. LEAFY target genes reveal floral regulatory logic, cis motifs, and a link to biotic stimulus response. Dev Cell. 2011;20:430–443. doi:10.1016/j.devcel.2011.03.019. 21497757

[18] Blazquez MA, Green R, Nilsson O, Sussman MR, Weigel D. Gibberellins promote flowering of *Arabidopsis* by activating the *LEAFY* promoter. Plant Cell. 1998;10:791–800. doi:10.1105/tpc.10.5.791. 9596637 PMC144373

[19] Navarro L, Bari R, Achard P, Lisón P, Nemri A, Harberd NP, Jones JDG. DELLAs control plant immune responses by modulating the balance of jasmonic acid and salicylic acid signaling. Curr Biol. 2008;18:650–655. doi:10.1016/j.cub.2008.03.060. 18450451

[20] Lyons R, Rusu A, Stiller J, Powell J, Manners JM, Kazan K. Investigating the association between flowering time and defense in the *Arabidopsis thaliana-Fusarium oxysporum* interaction. PloS One. 2015;10:e0127699. doi:10.1371/journal.pone.0127699. 26034991 PMC4452756

[21] Park DH, Somers DE, Kim YS, Choy YH, Lim HK, Soh MS, Kim HJ, Kay SA, Nam HG. Control of circadian rhythms and photoperiodic flowering by the *Arabidopsis* GIGANTEA gene. Science. 1999;285:1579–1582. doi:10.1126/science.285.5433.1579. 10477524

[22] Fowler S, Thomashow MF. *Arabidopsis* transcriptome profiling indicates that multiple regulatory pathways are activated during cold acclimation in addition to the CBF cold response pathway. Plant Cell. 2002;14:1675–1690. doi:10.1105/tpc.003483. 12172015 PMC151458

[23] Martin-Tryon EL, Kreps JA, Harmer SL. GIGANTEA acts in blue light signaling and has biochemically separable roles in circadian clock and flowering time regulation. Plant Physiol. 2007;143:473–486. doi:10.1104/pp.106.088757. 17098855 PMC1761957

[24] Mishra P, Panigrahi KC. GIGANTEA - an emerging story. Front Plant Sci. 2015;6:8. doi:10.3389/fpls.2015.00008. 25674098 PMC4306306

[25] Cao S, Ye M, Jiang S. Involvement of GIGANTEA gene in the regulation of the cold stress response in *Arabidopsis*. Plant Cell Rep. 2005;24:683–690. doi:10.1007/s00299-005-0061-x. 16231185

[26] Fornara F, de Montaigu A, Sánchez-Villarreal A, Takahashi Y, Ver Loren van Themaat E, Huettel B, Davis SJ, Coupland G. The GI – CDF module of *Arabidopsis* affects freezing tolerance and growth as well as flowering. Plant J. 2015;81:695–706. doi:10.1111/tpj.12759. 25600594 PMC5006856

[27] Kurepa J, Smalle J, Van Montagu M, Inzé D. Oxidative stress tolerance and longevity in *Arabidopsis*: the late-flowering mutant *gigantea* is tolerant to paraquat. Plant J. 1998;14:759–764. doi:10.1046/j.1365-313x.1998.00168.x. 9681039

[28] Riboni M, Galbiati M, Tonelli C, Conti L. GIGANTEA enables drought escape response via Abscisic acid-dependent activation of the florigens and SUPPRESSOR OF OVEREXPRESSION OF CONSTANS. Plant Physiol. 2013;162:1706–1719. doi:10.1104/pp.113.217729. 23719890 PMC3707542

[29] Kim WY, Ali Z, Park HJ, Park SJ, Cha JY, Perez-Hormaeche J, Quintero FJ, Shin G, Kim MR, Qiang Z, et al. Release of SOS2 kinase from sequestration with GIGANTEA determines salt tolerance in *Arabidopsis*. Nat Commun. 2013;4:1352. doi:10.1038/ncomms2357. 23322040

[30] Dodds PN, Rathjen JP. Plant immunity: towards an integrated view of plant-pathogen interactions. Nat Rev Genet. 2010;11:539–548. doi:10.1038/nrg2812. 20585331

[31] Macho AP, Zipfel C. Targeting of plant pattern recognition receptor-triggered immunity by bacterial type-III secretion system effectors. Curr Opin Microbiol. 2015;23:14–22. doi:10.1016/j.mib.2014.10.009. 25461568

[32] Cui H, Tsuda K, Parker JE. Effector-triggered immunity: from pathogen perception to robust defense. Annu Rev Plant Biol. 2015;66:487–511. doi:10.1146/annurev-arplant-050213-040012. 25494461

[33] Tsuda K, Somssich IE. Transcriptional networks in plant immunity. New Phytol. 2015;206:932–947. doi:10.1111/nph.13286. 25623163

[34] Zhou N, Tootle TL, Tsui F, Klessig DF, Glazebrook J. PAD4 functions upstream from salicylic acid to control defense responses in *Arabidopsis*. Plant Cell. 1998;10:1021–1030. doi:10.1105/tpc.10.6.1021. 9634589 PMC144042

[35] Wagner S, Stuttmann J, Rietz S, Guerois R, Brunstein E, Bautor J, Niefind K, Parker J. Structural basis for signaling by exclusive EDS1 heteromeric complexes with SAG101 or PAD4 in plant innate immunity. Cell Host Microbe. 2013;14:619–630. doi:10.1016/j.chom.2013.11.006. 24331460

[36] Rietz S, Stamm A, Malonek S, Wagner S, Becker D, Medina-Escobar N, Corina Vlot A, Feys BJ, Niefind K, Parker JE, et al. Different roles of ENHANCED DISEASE SUSCEPTIBILITY1 (EDS1) bound to and dissociated from PHYTOALEXIN DEFICIENT4 (PAD4) in *Arabidopsis* immunity. New Phytol. 2011;191:107–119. doi:10.1111/j.1469-8137.2011.03675.x. 21434927

[37] Seyfferth C, Tsuda K. Salicylic acid signal transduction: the initiation of biosynthesis, perception and transcriptional reprogramming. Front Plant Sci. 2014;5:697. doi:10.3389/fpls.2014.00697. 25538725 PMC4260477

[38] Fu ZQ, Dong X. Systemic acquired resistance: turning local infection into global defense. Annu Rev Plant Biol. 2013;64:839–863. doi:10.1146/annurev-arplant-042811-105606. 23373699

[39] Zhang X, Chen S, Mou Z. Nuclear localization of NPR1 is required for regulation of salicylate tolerance, *ISOCHORISMATE SYNTHASE1* expression and salicylate accumulation in *Arabidopsis*. J Plant Physiol. 2010;167:144–148. doi:10.1016/j.jplph.2009.08.002. 19716624

[40] Coates ME, Beynon JL. *Hyaloperonospora arabidopsidis* as a pathogen model. Annu Rev Phytopathol. 2010;48:329–345. doi:10.1146/annurev-phyto-080508-094422. 19400636

[41] Bartsch M, Gobbato E, Bednarek P, Debey S, Schultze JL, Bautor J, Parker JE . Salicylic acid-independent ENHANCED DISEASE SUSCEPTIBILITY1 signaling in *Arabidopsis* immunity and cell death is regulated by the monooxygenase FMO1 and the Nudix hydrolase NUDT7. Plant Cell. 2006;18:1038–1051. 16531493 10.1105/tpc.105.039982 PMC1425861

[42] Cui H, Gobbato E, Kracher B, Qiu J, Bautor J, Parker JE. A core function of EDS1 with PAD4 is to protect the salicylic acid defense sector in *Arabidopsis* immunity. New Phytol. 2017;213:1802–1817. doi:10.1111/nph.14302. 27861989

[43] Joglekar S, Suliman M, Bartsch M, Halder V, Maintz J, Bautor J, Zeier J, Parker JE, Kombrink E. Chemical activation of EDS1/PAD4 signaling leading to pathogen resistance in *Arabidopsis*. Plant Cell Physiol. 2018;59:1592–1607. doi:10.1093/pcp/pcy106. 29931201

[44] Mishina TE, Zeier J. The Arabidopsis flavin-dependent monooxygenase FMO1 is an essential component of biologically induced systemic acquired resistance. Plant Physiol. 2006;141:1666–1675. doi:10.1104/pp.106.081257. 16778014 PMC1533925

[45] Chen Y-C, Holmes EC, Rajniak J, Kim J-G, Tang S, Fischer CR, Mudgett MB, Sattely ES. N-hydroxy-pipecolic acid is a mobile metabolite that induces systemic disease resistance in Arabidopsis. Proc Natl Acad Sci USA. 2018;115:E4920–E9. doi:10.1073/pnas.1805291115. 29735713 PMC6003486

[46] Hartmann M, Zeier T, Bernsdorff F, Reichel-Deland V, Kim D, Hohmann M, Scholten N, Schuck S, Bräutigam A, Hölzel T, et al. Flavin monooxygenase-generated N-hydroxypipecolic acid is a critical element of plant systemic immunity. Cell. 2018;173:456–69. e16. doi:10.1016/j.cell.2018.02.049. 29576453

[47] Feys BJ, Wiermer M, Bhat RA, Moisan LJ, Medina-Escobar N, Neu C, Cabral A, Parker JE. *Arabidopsis* SENESCENCE-ASSOCIATED GENE101 Stabilizes and Signals within an ENHANCED DISEASE SUSCEPTIBILITY1 Complex in Plant Innate Immunity. Plant Cell. 2005;17(9):2601–2613. doi:10.1105/tpc.105.033910. 16040633 PMC1197438

[48] Cabral A, Stassen JH, Seidl MF, Bautor J, Parker JE, Van den Ackerveken G. Identification of *Hyaloperonospora arabidopsidis* transcript sequences expressed during infection reveals isolate-specific effectors. PLoS One. 2011;6:e19328. doi:10.1371/journal.pone.0019328. 21573066 PMC3090399

[49] Aarts N, Metz M, Holub E, Staskawicz BJ, Daniels MJ, Parker JE. Different requirements for EDS1 and NDR1 by disease resistance genes define at least two R gene-mediated signaling pathways in *Arabidopsis*. Proc Natl Acad Sci USA. 1998;95:10306–10311. doi:10.1073/pnas.95.17.10306. 9707643 PMC21504

[50] Ó’Maoiléidigh DS, van Driel AD, Singh A, Sang Q, Le Bec N, Vincent C, de Olalla EBG, Vayssières A, Romera Branchat M, Severing E, et al. Systematic analyses of the *MIR172* family members of *Arabidopsis* define their distinct roles in regulation of APETALA2 during floral transition. PLoS Biol. 2021;19:e3001043. 33529186 10.1371/journal.pbio.3001043 PMC7853530

[51] Kaufmann K, Muino JM, Østerås M, Farinelli L, Krajewski P, Angenent GC. Chromatin immunoprecipitation (ChIP) of plant transcription factors followed by sequencing (ChIP-SEQ) or hybridization to whole genome arrays (ChIP-CHIP). Nat Protoc. 2010;5:457–472. doi:10.1038/nprot.2009.244. 20203663

[52] Huq E, Tepperman JM, Quail PH. GIGANTEA is a nuclear protein involved in phytochrome signaling in *Arabidopsis*. Proc Natl Acad Sci U S A. 2000;97:9789–9794. doi:10.1073/pnas.170283997. 10920210 PMC16943

[53] Botella MA, Parker JE, Frost LN, Bittner-Eddy PD, Beynon JL, Daniels MJ, Holub EB, Jones JDG. Three genes of the Arabidopsis RPP1 complex resistance locus recognize distinct peronospora parasitica avirulence determinants. Plant Cell. 1998;10:1847–1860. doi:10.1105/tpc.10.11.1847. 9811793 PMC143951

[54] Asai S, Rallapalli G, Piquerez SJ, Caillaud MC, Furzer OJ, Ishaque N, Wirthmueller L, Fabro G, Shirasu K, Jones JDG, et al. Expression profiling during *Arabidopsis*/downy mildew interaction reveals a highly-expressed effector that attenuates responses to salicylic acid. PLoS Pathog. 2014;10:e1004443. doi:10.1371/journal.ppat.1004443. 25329884 PMC4199768

[55] Asai S, Yoshioka H. Nitric oxide as a partner of reactive oxygen species participates in disease resistance to necrotrophic pathogen *Botrytis cinerea* in *Nicotiana benthamiana*. Mol Plant Microbe Interact. 2009;22:619–629. doi:10.1094/MPMI-22-6-0619. 19445587

[56] Fowler S, Lee K, Onouchi H, Samach A, Richardson K, Morris B, Coupland G, Putterill, J, . GIGANTEA: a circadian clock‐controlled gene that regulates photoperiodic flowering in *Arabidopsis* and encodes a protein with several possible membrane‐spanning domains. The EMBO J. 1999;18:4679–4688. doi:10.1093/emboj/18.17.4679. 10469647 PMC1171541

[57] Nohales MA, Liu W, Duffy T, Nozue K, Sawa M, Pruneda-Paz JL, Maloof JN, Jacobsen SE, Kay SA. Multi-level modulation of light signaling by GIGANTEA regulates both the output and pace of the circadian clock. Dev Cell. 2019;49:840–51. e8. doi:10.1016/j.devcel.2019.04.030. 31105011 PMC6597437

[58] Giri MK, Singh N, Banday ZZ, Singh V, Ram H, Singh D, Chattopadhyay S, Nandi AK. GBF1 differentially regulates CAT2 and PAD4 transcription to promote pathogen defense in *Arabidopsis thaliana*. Plant J. 2017;91:802–815. doi:10.1111/tpj.13608. 28622438

[59] Kundu P, Sahu R. GIGANTEA confers susceptibility to plants during spot blotch attack by regulating salicylic acid signalling pathway. Plant Physiol Biochem. 2021. doi:10.1016/j.plaphy.2021.02.006. 34399204

[60] Gould PD, Locke JC, Larue C, Southern MM, Davis SJ, Hanano S, Moyle R, Milich R, Putterill J, Millar AJ, et al. The molecular basis of temperature compensation in the *Arabidopsis* circadian clock. Plant Cell. 2006;18:1177–1187. doi:10.1105/tpc.105.039990. 16617099 PMC1456873

[61] Araki T, Komeda Y. Analysis of the role of the late‐flowering locus, GI, in the flowering of *Arabidopsis thaliana*. Plant J. 1993;3:231–239. doi:10.1046/j.1365-313X.1993.t01-15-00999.x.

[62] Häffner E, Konietzki S, Diederichsen E. Keeping control: the role of senescence and development in plant pathogenesis and defense. Plants. 2015;4:449–488. doi:10.3390/plants4030449. 27135337 PMC4844401

[63] Wiermer M, Feys BJ, Parker JE. Plant immunity: the EDS1 regulatory node. Curr Opin Plant Biol. 2005;8:383–389. doi:10.1016/j.pbi.2005.05.010. 15939664

