# Supplementary material for: GIGANTEA regulates PAD4 transcription to promote pathogen defense against Hyaloperonospora arabidopsidis in Arabidopsis thaliana
Source: Plant Signal Behav. 2022 Apr 4;17(1):2058719. doi: 10.1080/15592324.2022.2058719 (PMC8986176; doi:10.1080/15592324.2022.2058719)
Supplement: Supplemental Material [file KPSB_A_2058719_SM6486.zip › Revised_supplementary_figures.docx]

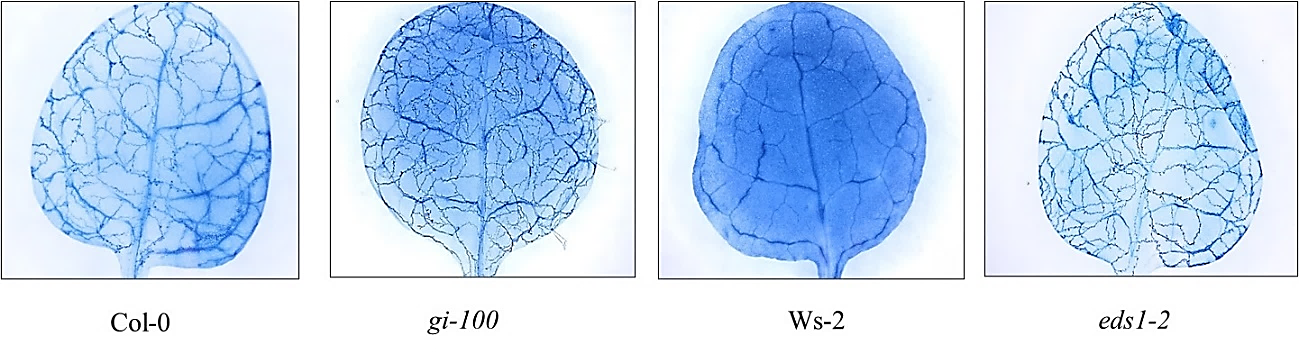


**Supplementary Figure S1: Conidiospores phenotyping in *gi-100* mutant.** Evaluation of Noco2 infection was done by trypan blue staining of the conidiospores in *gi-100* mutant (experimental) and Col-0 (wild-type) at 5 dpi. Ws-2 plants which show resistance to Noco2 pathogen was used as a negative control and *eds1-2* which shows high susceptibility to Noco2 infection was used as a positive control.


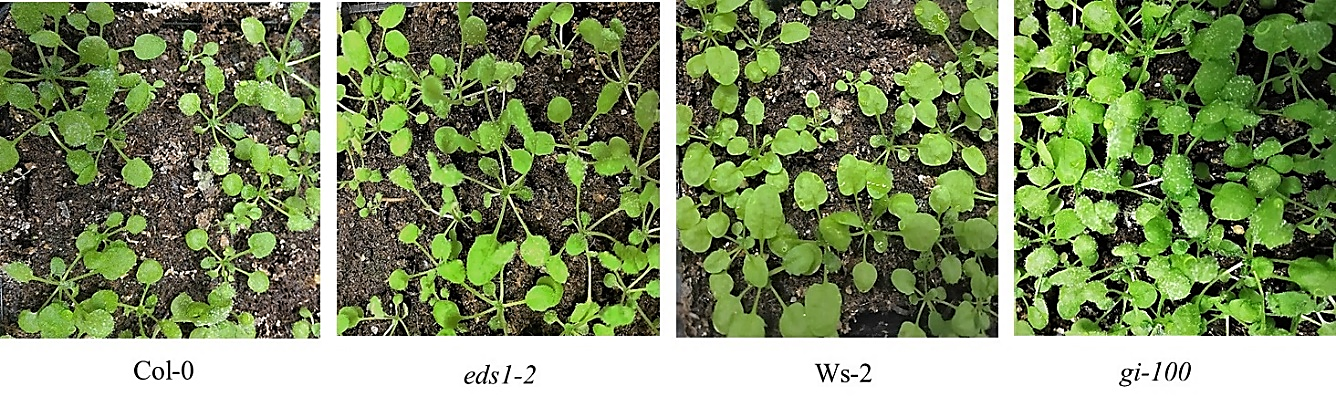


**Supplementary Figure S2: Plant phenotype showing *Hpa* Noco2 infection in Col-0, *eds1-2,* Ws-2, and *gi-100* plants after 6 d of inoculation**. For *Hpa* infections, conidiospore suspensions (5×10^4^ conidiospores/ml) were sprayed on 2-week-old *Arabidopsis* seedlings grown on potting soil in a growth chamber at 22°C with 8 h of light. Plants were then allowed to dry for 1 h and kept at 100% RH for 24 h with 8 h light at 22°C. Plants were then moved to ∼75% RH for infection to progress. Photographs of the indicated genotypes were taken after 6 d of infection with Noco2.

**Supplementary Figure S3: Graph showing conidiospores count (*10^4^/g) after 6 d of Noco2 infection in Col-0, *eds1-2*, Ws-2, *gi-100,* and *gi-2****.* Plants were grown on potting soil in a growth chamber at 22°C with 8 h of light and relative humidity of 75%. Infection with *Hpa* Noco2 (10^4^ conidiospores per milliliter), which is virulent on Col-0, was done and the appearance of conidiospores was scored 6 d later using a hemocytometer. Three biological replicates were used for the experiments. To test for significance among the dataset, a one-way analysis of variance (ANOVA) followed by Sidak’s multiple comparisons test was performed using GraphPad prism software at *p<0.05. ** p<0.01, *** p<0.001.
