# Supplementary material for: GIGANTEA regulates PAD4 transcription to promote pathogen defense against Hyaloperonospora arabidopsidis in Arabidopsis thaliana
Source: Plant Signal Behav. 2022 Apr 4;17(1):2058719. doi: 10.1080/15592324.2022.2058719 (PMC8986176; doi:10.1080/15592324.2022.2058719)
Supplement: Supplemental Material [file KPSB_A_2058719_SM6486.zip › Supplementary_Table.docx]

**Supplementary Table 1: List of primers used for RT-qPCR**

| **Gene name** | **Forward Primer (5'-3')** | **Reverse Primer (5'-3')** |
| --- | --- | --- |
| ***PR1*** | GGTTAGCGAGAAGGCTAACTAC | CATCCGAGTCTCACTGACTTTC |
| ***PBS3*** | CACAAATTTCGCTGGCTTGT | TCCTTCTCCTCTCCCTCTTTG |
| ***ICS1*** | TAACGAGAACGGAAACGGAAA | GGATCAAGGTCACGGAAGAAA |
| ***PAD4*** | CCGCACTTTGGCTTCTATCT | GAGGTGGAGAGAGATTGGTTTC |
| ***FMO1*** | TGCCTTTATACAGGGGAACA | TGGAAATGCAATGACGTTTG |
| ***GIGANTEA*** | GCTTCTCGAGGATCTGGTAAAC | GTCAGCGTAGCAGTCTCATATC |
| ***ACTIN1*** | CGATGAAGCTCAATCCAAACGA | CAGAGTCGAGCACAATACCG |

**Supplementary Table 2: List of primers sequences used in ChIP-qPCR**

| **Gene name** | **Forward Primer (5'-3')** | **Reverse Primer (5'-3')** |
| --- | --- | --- |
| ***PAD4*ChIPexon** | GTCGATTCGAGACGAGTGAG | GATCTTTATACTCCCGTTGC |
| ***PAD4*ChIPintron** | CACCTACCCCAATAGGCAAT | GGTGTCAAAAATACCAATGACG |
| ***PAD4*ChIPpromoter** | TGATTTGTTACACCGTTTTTCG | TCACCACATGTTATCGTCTCC |
